# Flash NanoPrecipitation as an Agrochemical Nanocarrier Formulation Platform: Phloem Uptake and Translocation after Foliar Administration

**DOI:** 10.1021/acsagscitech.3c00204

**Published:** 2023-10-17

**Authors:** Kurt Ristroph, Yilin Zhang, Valeria Nava, Jonas Wielinski, Hagay Kohay, Andrew M. Kiss, Juergen Thieme, Gregory V. Lowry

**Affiliations:** † Department of Civil and Environmental Engineering, Carnegie Mellon University, Pittsburgh, Pennsylvania 15213-3815, United States; ‡ NSLS-II, Brookhaven National Laboratory, Upton, New York 11973-5000, United States

**Keywords:** Flash NanoPrecipitation, nanocarrier, foliar uptake, agrochemicals, phloem loading, sustainable agriculture

## Abstract

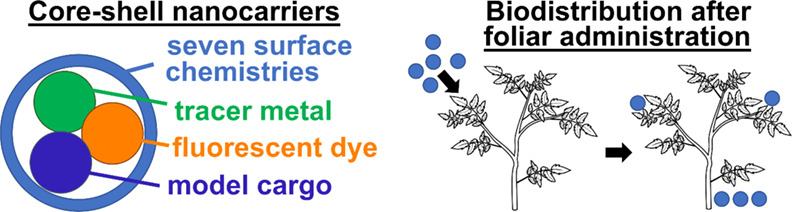

The increasing severity of pathogenic and environmental stressors that negatively affect plant health has led to interest in developing next-generation agrochemical delivery systems capable of precisely transporting active agents to specific sites within plants. In this work, we adapt Flash NanoPrecipitation (FNP), a scalable nanocarrier (NC) formulation technology used in the pharmaceutical industry, to prepare organic core–shell NCs and study their efficacy as foliar or root delivery vehicles. NCs ranging in diameter from 55 to 200 nm, with surface zeta potentials from −40 to +40 mV, and with seven different shell material properties were prepared and studied. Shell materials included synthetic polymers poly(acrylic acid), poly(ethylene glycol), and poly(2-(dimethylamino)ethyl methacrylate), naturally occurring compounds fish gelatin and soybean lecithin, and semisynthetic hydroxypropyl methylcellulose acetate succinate (HPMCAS). NC cores contained a gadolinium tracer for tracking by mass spectrometry, a fluorescent dye for tracking by confocal microscopy, and model hydrophobic compounds (alpha tocopherol acetate and polystyrene) that could be replaced by agrochemical payloads in subsequent applications. After foliar application onto tomato plants with Silwet L-77 surfactant, internalization efficiencies of up to 85% and NC translocation efficiencies of up to 32% were observed. Significant NC trafficking to the stem and roots suggests a high degree of phloem loading for some of these formulations. Results were corroborated by confocal microscopy and synchrotron X-ray fluorescence mapping. NCs stabilized by cellulosic HPMCAS exhibited the highest degree of translocation, followed by formulations with a significant surface charge. The results from this work indicate that biocompatible materials like HPMCAS are promising agrochemical delivery vehicles in an industrially viable pharmaceutical nanoformulation process (FNP) and shed light on the optimal properties of organic NCs for efficient foliar uptake, translocation, and delivery.

## Introduction

Environmental stressors and a growing population necessitate the development of innovative materials, practices, and technologies across the agricultural sector to maintain and improve crop yields in the future.^1,2^ One area of research interest is improving the efficacy of existing agrochemicals by designing novel dosage forms that can both be easily administered and promote precision delivery to target tissues in plants. Foliar delivery of bioactive agents to plants is a highly desirable administration route but is a historically inefficient method of promoting agrochemical internalization or trafficking of agrochemicals to specific locations in plant tissue.^3^ Most agrochemicals, when applied foliarly in the form of an aqueous dispersion, are poorly internalized into leaves and do not transit efficiently to other organs, limiting their ability to affect biological activity in the target organism.^4^ Hydrophilic agents are unable to penetrate the waxy cuticular layer on leaves,^5^ and hydrophobic agents are usually dispersed in suspensions with macroscopic particulates, which are too large for internalization by direct penetration or stomatal flooding.^6,7,4^ Technology to improve agrochemical uptake and systemic distribution to vasculature, roots, other leaves, or combinations of these is therefore highly desirable in the development of next-generation fertilizers, fungicides, pesticides, and other bioactives.^8−11^

To this end, formulating agrochemicals into nanoparticles or nanocarriers (NCs) is a growing area of interest,^7,12^ but several obstacles have persisted in this research. The first is that most of the nanoparticles studied in the agrochemical delivery literature are metallic and incapable of encapsulating primarily organic active ingredients (AI) for delivery in plants.^7^ While some metal nanoparticles exhibit useful bioactive characteristics such as antibacterial activity (e.g., Ag-based nanoparticles^13^) and others may serve as a source of micronutrient metals (e.g., Fe^14^ and S^15^) or both (e.g., Cu-based NPs^16^), their use as delivery vehicles is limited to the delivery of their constituent metals or surface-bound organic molecules. The second is that the impact of the nanoparticle size, charge, and surface properties on their uptake and transport is not fully understood.^7,9^ Two identical metal nanoparticle formulations with surface-bound payloads of widely differing chemistries would not be expected to translocate identically in a plant, meaning that optimizing uptake and translocation would have to be done on a case-by-case basis, which is expensive and time-consuming. The third is that nanomaterial manufacturability at a large scale needed for agrochemicals remains a challenge. The development of cost-effective, scalable, organic, and biodegradable NCs with a core–shell geometry could overcome these barriers. Particles coated by a dense hydrophilic surface shell layer with properties independent of the payload core could be attractive agrochemical delivery vehicles. This approach can enable the delivery of a range of organic AI payloads with the same size and shell chemistry that have been optimized for uptake and transport to selected locations in the plant.

Flash NanoPrecipitation (FNP) is a continuous, scalable NC formulation process originally developed to encapsulate strongly hydrophobic drugs into core–shell NCs stabilized by block copolymers.^17,18^ FNP has been implemented at the industrial scale in the pharmaceutical industry and is the technology Pfizer uses to formulate lipid NPs in its SARS-CoV-2 vaccine.^19^ The size of FNP NCs is tunable from 35 to 400 nm,^20^ and core loadings, i.e., the mass of core per mass of total NC, of 50–70 wt % are typical, and loadings up to 90% have been reported.^21,22^ The cores of FNP NCs usually consist entirely of the payload(s) to be delivered, meaning that a given formulation could consist of up to 70% payload by mass. This is significantly higher than the approach that coats a metal particle surface with an AI and reduces the total amount of material—payload (core) plus any inactive excipients (shell)—that must be applied and taken up by the plants in order to achieve a therapeutic dose, reducing the cost and material burden. FNP also produces NCs with a dense surface layer (densities up to 1.5 polymer chains per nm^2^ have been reported for block copolymer-stabilized NCs^20^) that prevents the chemistry of the core components from contributing to the overall carrier surface properties.

Two recent advancements in FNP make the technology an attractive option for nanoformulating agrochemicals. The first enables the encapsulation of ionizable hydrophilic molecules,^23−28^ enabling encapsulation of both hydrophobic and hydrophilic AIs, and the second replaces high-cost block copolymer stabilizers with low-cost alternatives such as lecithin,^29^ gelatin,^30^ a combination of the corn protein zein with the milk protein byproduct casein,^31^ and hydroxypropyl methylcellulose acetate succinate (HPMCAS),^24,31−34^ a semisynthetic cellulose derivative used in the pharmaceutical industry. The result is a platform technology for manufacturing, at large scales and low per-unit costs, NCs capable of encapsulating a wide range of payloads.

In this work, we study the application of NCs prepared by FNP as foliar delivery agents. Specifically, we explore the large design space of NC sizes and surface chemistries that are accessible by FNP to identify candidate NCs that can provide efficient uptake into leaves and high phloem loading and translocation efficiency. The core–shell nature of FNP NCs is significant because surface chemistry is independent of the core material, meaning that two different NC formulations of the same size with the same surface stabilizer and different encapsulated core payloads would be expected to translocate identically. Incorporation of an AI into the core is not expected to affect the shell properties or the uptake and translocation properties. NC uptake into tomato leaves and translocation following foliar application were studied by confocal microscopy and mass spectrometry using a suite of multifunctional NCs: NC cores contained encapsulated Gd tracer metal for detection by mass spectrometry and X-ray fluorescence mapping as well as a fluorescent dye for confocal microscopy. The findings indicated that some formulations provide high uptake efficiency (up to 84%) and high translocation (up to 32%) away from the applied leaf to other plant organs.

## Materials and Methods

### Materials

Poly(styrene)_1.8k_-*b*-poly(ethylene glycol)_5k_ (PS-*b*-PEG), poly(styrene)_1.8k_-*b*-poly(acrylic acid)_6k_ (PS-*b*-PAA), poly(styrene)_9k_-*b*-poly(*n*,*n*-dimethylaminoethyl methacrylate)_4k_ (PS-*b*-PDMAEMA), and poly(styrene)_1.8k_ homopolymer (PS) were purchased from PolymerSource (Dorval, Quebec, CA). Lecithin, hydrogen peroxide, nitric acid trace metal grade, tetrahydrofuran (THF), sodium acetate, potassium chloride (KCl), and 6–8 kDa MWCO dialysis tubing were purchased from Fisher Scientific (Waltham, MA, USA). Gelatin from cold water fish skin, α-tocopherol acetate, and 1,4,8,11,15,18,22,25-octabutoxy-29*H*,31*H*-phthalocyanine (“762 dye”) were purchased from Sigma-Aldrich (St. Louis, MO, USA). HPMCAS126 (HPMCAS) was a gift from Dow Chemical. Hydrochloric acid (HCl) was purchased from VWR and used to adjust the pH of the antisolvent buffer used during NC formulation to 5.5. Sodium hydroxide (NaOH) was purchased from Honeywell. Tissue-Tek O.C.T. Compound was purchased from Epredia. Hydrophobic gadolinium oxide colloids (∼2 nm) dispersed in THF were a gift from Dr. Robert Prud’homme’s Lab at Princeton University. Roma tomato seeds were purchased from W. Atlee Burpee & Co (Warminster, PA, USA).

### NC Formulation and Characterization

**Figure 1:**
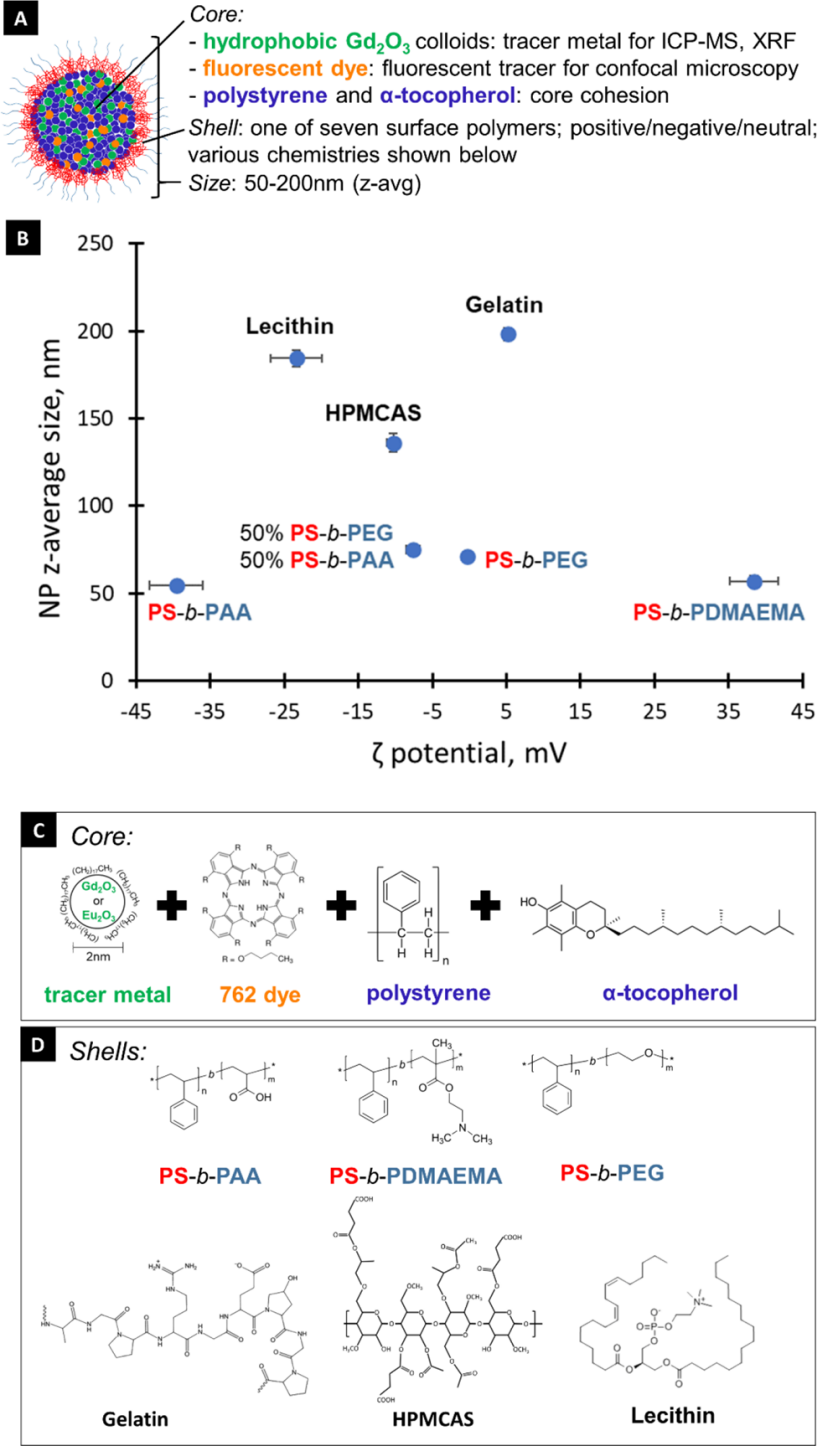
(A) Schematic of core–shell NCs. (B) NC DLS size (measured in DI water) and zeta potential (measured at neutral pH in 15 mM KCl) for the seven formulations prepared for this study. Structures of the NC (C) core and (D) shell components.

NCs were formulated using FNP in either a confined impinging jet mixer or multi-inlet vortex mixer, as described previously.^17^ In brief, hydrophobic core materials were dissolved in THF (0.5 mL), then rapidly mixed against an aqueous antisolvent stream (0.5 mL), and collected in a quench water bath (4 mL). The concentrations in the feed streams of all formulations tested here are listed in Table S1. In all formulations, NC cores consisted of hydrophobic Gd or Eu colloids (tracer metals used for mass spectrometry), 1,4,8,11,15,18,22,25-octabutoxy-29*H*,31*H*-phthalocyanine dye (“762 dye,” fluorescent dye used for confocal microscopy), and PS and α-tocopherol acetate (hydrophobic agents used to promote core cohesion). Surface stabilizers tested included PS-*b*-PAA, PS-*b*-PEG, PS-*b*-PDMAEMA, gelatin, lecithin, HPMCAS, and a 50/50 w/w mixture of PS-*b*-PAA and PS-*b*-PEG (Figure 1). Following FNP, residual organic solvent was removed (see Supplemental Methods), and the resulting NCs were used for all subsequent tests.

In FNP, rapid turbulent mixing between the streams achieves homogeneity on the order of 3–5 ms, which is then followed by diffusion-limited aggregation of hydrophobic nanoparticle components by hydrophobic interactions to form the NC core.^20,35^ NC stabilization is achieved by the adsorption of a dense layer of the amphiphilic surface molecule, which arrests nucleation and provides colloidal stability in water. The quench bath reduces the volume fraction of THF in the final suspension to 10%, which stabilizes particles against THF-driven Ostwald ripening until the particles can be dialyzed or rinsed to remove residual organic solvent.

The NC size, polydispersity index, and zeta potential were measured by using a Malvern Zetasizer Nano (Malvern Instruments). NCs were diluted 10-fold in deionized water prior to dynamic light scattering (DLS) size measurement to reduce multiple scattering, and measurements were performed in triplicate. NCs were diluted 10-fold in 15 mM KCl prior to zeta potential measurement (neutral pH). NC metal content was quantified by inductively coupled plasma mass spectrometry (ICP-MS). To 0.1 mL of NC suspension was added 0.5 mL of nitric acid, and then 30 min later, 9.4 mL of deionized water was added. The metal content in each digested sample was then measured using an Agilent 7700 ICP-MS instrument and matched with expected values (based on the feed concentration of Gd), indicating complete digestion.

After a primary rinse to remove the organic solvent and the unbound metal (see SI section “Organic Solvent Removal”), 2 mL of NC suspension was dialyzed (6–8k MWCO dialysis tubing, Thermo Fisher Scientific) against 250 mL of simulated apoplastic fluid (SAF) for 72 h to confirm that the encapsulated tracer metal did not leach from NCs over the time scale of the subsequent tests. Aliquots of the bulk SAF were removed over time, and Gd concentration was quantified by ICP-MS. The leached metal was calculated by dividing the mass of the metal in 2 mL of NC suspension prior to dialysis by the mass of the metal detected in the bulk dialysis SAF. The SAF composition is described in the Supplemental Methods.

### NC Foliar Dosing

NCs were dosed foliarly on 4-week old Roma tomato seedlings grown hydroponically in quarter-strength Hoagland’s hydroponic solution, as described previously.^10^ In brief, 1 mL of NC suspension was added to 1 μL of Silwet L-77 surfactant, then 20–85 μL (depending on the subsequent test) of the NC-Silwet suspension was carefully pipetted across the adaxial side of the true leaf second from the bottom of the seedling. The (0.1 vol %) Silwet surfactant in the suspension promoted rapid spreading of the fluid across the leaf.

### NC Translocation

72 h after foliar NC application, plants were harvested and sectioned into six sections, as described previously: upper leaves (younger than dosed leaf), lower leaves (older than dosed leaf), dosed leaf, stem, roots, and Hoagland solution.^10^ Plant tissue sections were dried in an oven for 48 h at 95 °C, digested with a 1:2 v/v mixture of hydrogen peroxide and 70% nitric acid solution for at least 24 h, heated to 95 °C for 1 h using a VWR digital heat block to ensure complete digestion, diluted 10-fold with deionized water, and filtered using 0.45 μm PTFE hydrophobic BioExcell syringe filters (25 mm). An aliquot of 0.25 mL of 70% nitric acid was added to 4.75 mL of Hoagland’s solution, and the solution was then filtered using the same filter listed above. Gd content in each section was quantified by ICP-MS using an Agilent 7700 ICP mass spectrometer. Seven formulations were assessed, as well as an undosed control and a control foliarly dosed with free gadolinium in the form of a gadolinium(III) chloride (7 ppm) Silwet solution. *N* = 5 plants were evaluated per NC formulation or control.

### NC Uptake

To quantify NC uptake into leaves, a combination of surface washing and hyperspectral imaging was used. To distinguish between NCs that were taken up into the leaf and those adsorbed outside of the leaf, dosed leaves (*n* = 5 leaves per formulation; formulations and controls were the same as listed above) were harvested 24 h after foliar NC application, placed in a 50 mL Falcon tube with 4.75 mL of water, and vigorously vortexed for 60 s to remove any surface-bound NCs. Next, the concentration of the metal in the leaves and wash water was quantified by ICP-MS. Leaves were removed from the wash water, then dried, and digested as described above. As above, 0.25 mL of 70% nitric acid was added to 4.75 mL of wash water. Gd content in both sections was quantified by ICP-MS. Statistical difference in the leaf-associated and water-associated metal between the Gd control and each of the NC formulations was calculated using Student’s *t*-analysis.

The above procedure alone could not unequivocally confirm NC uptake into leaves, only the mass of NCs that remained associated with leaves after the washing step—so hyperspectral imaging was used to determine whether NCs remained on the surface of the leaf. This procedure and its results are described in the Supporting Information.

### Confocal Microscopy

Fluorescent confocal microscopy was used to visualize the distribution of NCs internalized into leaf mesophyll. 24 h after foliar NC application, the dosed leaves were harvested; a small section was cut, mounted on a glass slide, and visualized on a Zeiss 880 confocal microscope. Chlorophyll was excited with a 633 nm laser, and emission was collected from 647 to 721 nm. NCs were excited with a 405 nm laser, and emission was collected from 441 to 499 nm. Leaf samples from undosed plants were also imaged and used for background autofluorescence subtraction.

## Results and Discussion

### NC Formulation and Characterization

Model NCs encapsulating Gd tracer metal and a fluorescent dye were successfully formed by using FNP (Figure 1A). Other core materials were α-tocopherol acetate and PS homopolymer, which were used here for hydrophobic core cohesion and core bulking. It is important to note that these particular model core compounds would be replaced with a bioactive material in an applied agrochemical nanoformulation, but the surrogate compounds used here for uptake and translocation testing are good analogues for those AIs. The range of *z*-average size and zeta potential of the seven model NC formulations is presented graphically in Figure 1B. The same data, also including polydispersity index, Gd content, core loading (*m*_core_/*m*_NC_), and Gd loss to SAF solution over 72 h, are shown in Table S2. NC size ranged from 55 to 200 nm by DLS, and zeta potential ranged from −40 to 40 mV and included NCs with a relatively neutral surface (5.2, −0.3 mV). For NPs produced by FNP, DLS measurements have been shown to be qualitatively similar to results obtained from population image analysis by transmission electron microscopy showing them to be spherical.^36^ Core loadings (combined mass of Gd colloids, vitamin E, 762 dyes, and polystyrene per mass of NC) are a good indicator for the amount of AI that could be encapsulated into the cores of different FNP particles made here. The core loadings were 25% (HPMCAS-stabilized NCs), 33% (lecithin- and gelatin-stabilized NCs), or 50% (other formulations), which are significantly higher than the 2–5% achieved by many NC formulations presented in drug delivery literature, and suggest the potential for encapsulating high amounts of AI per FNP particle.^37^ Gd loss from the core over 72 h of SAF dialysis was negligible or low (<1.4% in all cases except lecithin-stabilized NCs, where 7% of Gd was lost during dialysis). The incorporation of Gd_2_O_3_ particles into the core rather than Gd^3+^ contributes to their overall stability in the NC formulation.^38^ The reasons for the higher leaching of Gd from the lecithin-stabilized particles are not clear. The low mass of leached Gd indicates the suitability of using Gd quantification by ICP-MS to trace NC movement in plant tissues.

### NC Uptake into Leaf Mesophyll

**Figure 2:**
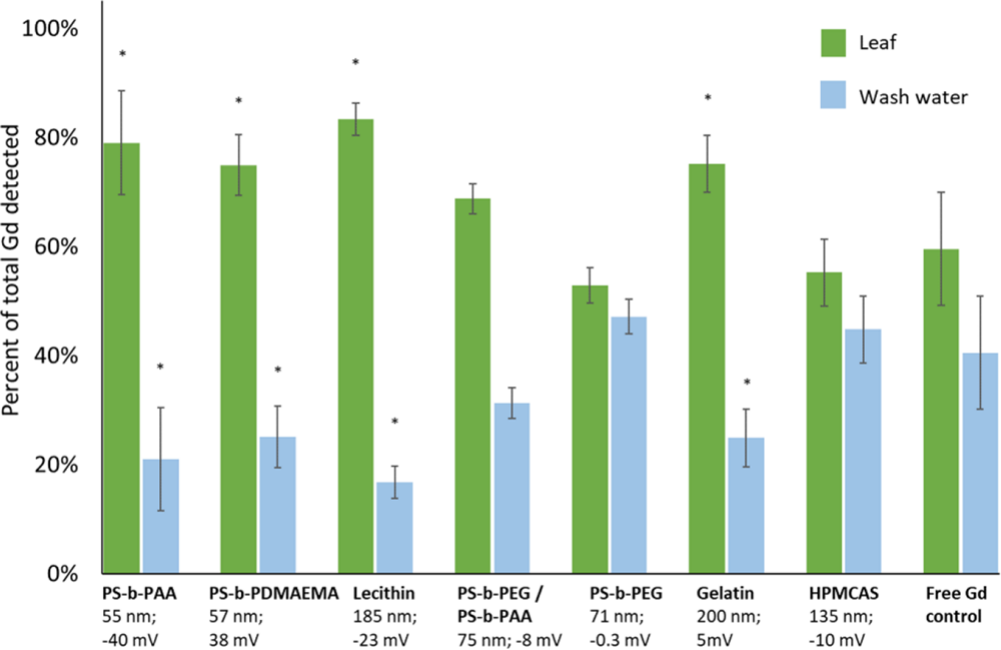
Rainfastness of applied NCs compared to the free Gd control. Fraction of Gd tracer metal detected in the leaf tissue (green) and wash water (blue) after leaves were vigorously vortexed in water to remove surface-bound NCs. Statistical difference from the free Gd control (*p* < 0.05) is denoted with an asterisk. Taken together with the hyperspectral images in Figure S2, these results suggest a high degree of NC internalization into the leaf mesophyll.

Rainfastness, i.e., the ability of the particles to remain on the leaf upon watering, is an important parameter affecting the efficacy of foliar applied agrochemicals. We approximated this property by vigorously washing the leaves after dosing them (Figure 2). For four of the seven formulations tested, NCs remained associated with the leaf tissue to a significantly higher extent than free Gd, averaging around 70%. This suggests that these NCs are strongly bound to the leaf surface or are being efficiently internalized into the tomato leaf mesophyll.

To determine if the NCs detected in the leaves after washing were strongly surface-bound or were taken up into the leaves, leaf surfaces were imaged by hyperspectral imaging.^8^ After washing, no NCs were detected on the leaf epidermis (Figure S2H), suggesting the source of leaf associated Gd in Figure 2 was NCs that had been taken up into the leaf mesophyll rather than NCs on the leaf exterior.

**Figure 3:**
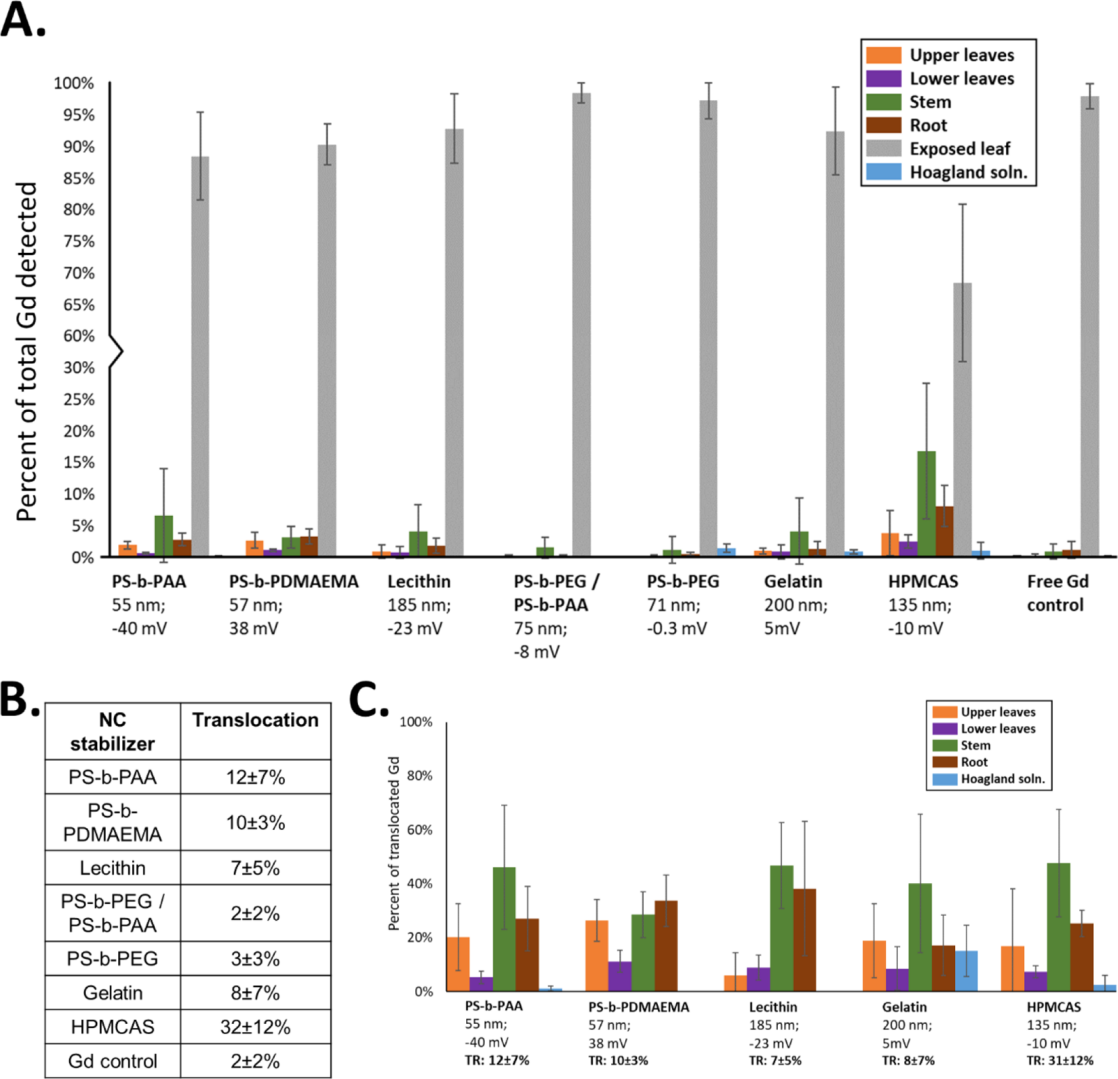
NC (A, B) translocation and (C) distribution in the tomato tissue 72 h after foliar dosing with NCs in 0.1 vol% Silwet. HPMCAS-coated NCs had the highest degree of translocation (32%). Anionic PS-*b*-PAA and cationic PS-*b*-PDMAEMA NCs exhibited approximately 11% translocation, with the majority of translocated Gd appearing in the stem and root tissue. Anionic lecithin-stabilized NCs translocated to a lesser degree. Neutral NCs exhibited limited translocation. Tissue distributions in (C) are only shown for formulations with greater than 5% overall translocation. These results suggest that surface charge is a more significant factor in NC translocation than size, but the identity of the surface molecules is the most important.

No NC signature was detected on the epidermis of leaves dosed with NCs and Silwet, regardless of washing (Figure S3G), even as soon as 2 h after dosing (Figure S2I). This suggests that the NCs are taken up in the leaf mesophyll relatively quickly. The precise rates of NC internalization and translocation remain largely unknown and will be the focus of future work. This rapid uptake into the leaf mesophyll is especially significant to note because the HPMCAS formulation, which exhibited the best translocation (Figure 3), appears in Figure 2 to exhibit similar uptake to free Gd. However, the total metal mass recovered from the dosed leaf and wash water in the test of HPMCAS-stabilized NCs was only ∼25% of the expected dose (Table S4). We hypothesize that a significant amount of NCs were internalized and translocated from the dosed leaf in the 24 h between dosing and washing, and this delay remains to be optimized.

It is notable that the results in Figure 2 do not appear to correlate with the NC size or surface charge. This suggests that one of the major mechanisms of NC uptake is Silwet-facilitated stomatal flooding since the smallest dimension of the stomatal aperture is on the order of 1 μm and the largest NCs prepared here are 200 nm in diameter. Previous work has measured the uptake and translocation of polymer macromolecules ranging between 5 and 35 nm in diameter^9^ or gold nanoparticles 3–50 nm in diameter with a surface-adsorbed polyvinylpyrrolidone layer.^11^ Both the studies concluded that in addition to promoting stomatal flooding, the presence of Silwet disturbed epidermis cells and allowed nanomaterials to be taken up into the leaf mesophyll through cuticle penetration. The NCs studied here are larger in size (40–200 nm in diameter), but the total absence of NCs detected on the epidermis in Figure S2G–I suggests that uptake also likely involves some cuticle penetration in addition to stomatal flooding.

### NC Translocation to Plant Tissue

Understanding how NC properties, such as size and surface charge, affect systemic translocation to plant tissue following uptake into the leaf mesophyll is critical in the development of an NC system for the precision delivery of agrochemicals. Some applications may call for systemic translocation, for example, treatments for phloem diseases such as HLB in citrus, while in others, high leaf uptake and low translocation may be desirable, for example, for treating or preventing fungal infections of leaf mesophyll or mitigating the effects of climate stress on photosynthesis.^39^

The amount of translation and the locations of translocated FNP particles depended on the shell properties (Figure 3 and Table S3), and several trends can be observed. First, NCs with higher surface charge (zeta potential >10 mV or ←10 mV) exhibited higher mobility than the three neutral NC formulations (zeta potential between −10 and 10 mV) and significantly higher mobility than Gd^3+^. Second, most translocated particles are in the stem after phloem loading. The HPMCAS-coated NCs exhibited the highest degree of translocation, with about 32% trafficked to other plant tissues (Figure 3A,B). Of the translocated HPMCAS-coated NCs, about 50% were detected in the plant stem, with another 25% detected in the roots, and 20% in the upper leaves (Figure 3C). NCs coated with anionic PS-*b*-PAA and cationic PS-*b*-PDMAEMA exhibited similar degrees of translocation to one another, with tissue distributions similar to those of the HPMCAS NCs. Anionic lecithin-coated NCs exhibited less overall translocation (approximately 7%), with the majority detected in the stem and root tissue. Neutral NCs coated with PS-*b*-PEG, PS-*b*-PEG + PS-*b*-PAA, or gelatin exhibited almost no movement in the plant and had translocation profiles indistinguishable from the free Gd control, which was not expected to efficiently translocate.^10^

It may be expected that smaller NCs would be translocated more efficiently than larger NCs and that charged vs neutral NCs may display different movement. These design rules are not yet firmly established for plants, and a relative weighting of the two factors is difficult to predict. The results presented here suggest that the NC surface charge is generally a more significant factor governing translocation than the NC size. The NCs coated with PS-*b*-PAA, PS-*b*-PDMAEMA, PS-*b*-PEG, and PS-*b*-PEG + PS-*b*-PAA had similar sizes (∼65 nm), but the NCs with a high magnitude of surface charge (either positive or negative) translocated to a much greater extent than the neutral NC formulations. The lecithin-coated NCs, which had a high surface charge, also translocated more than the neutral formulations. The PAA-coated NCs and lecithin-coated NCs had similar surface charges, and the smaller PAA-coated NCs translocated more than the larger lecithin NCs, in line with expectations around the effect of size.

The results in the preceding paragraph are consistent with the lipid exchange envelope penetration (LEEP) model or nanoparticle uptake into protoplasts proposed by Wong et al.^40^ Previous studies on the mechanism for nanomaterial transport in the plant tissue suggest that phloem loading is a prerequisite for systemic translocation following foliar administration. To load into a phloem, NCs must first pass the cell wall and then be internalized into mesophyll cells. The LEEP model predicts the second step of this process, i.e., uptake through the lipid bilayer of the mesophyll cells. The LEEP model suggests that the magnitude of NC surface charge, but not the sign, governs this uptake and that neutral NCs cannot be spontaneously taken up through plant lipid membranes. Our results, which show a similar translocation of anionic and cationic NCs with opposite surface charges, appear to follow this prediction of the model. However, differences with the LEEP model could also arise if the particles pass the cell wall at different rates due to their surface chemistry. Passage of particles through the cell wall is not considered in the LEEP model.

The HPMCAS-coated NC formulation is an outlier in this analysis. This formulation had the highest degree of translocation despite having both the third largest diameter (135 nm) of all NCs tested and a weakly anionic surface charge (−10 mV). These characteristics would suggest low cellular uptake and translocation, but the opposite was observed. This result underscores the point that particle surface charge is a proxy for the exact particle surface chemical composition, the latter of which appears to be the most significant factor in NC movement. HPMCAS is a semisynthetic cellulose derivative that may be more highly compatible with cellulosic cell walls than other materials tested as stabilizers. This could increase the amount of the NC that reaches the protoplast’s cell membrane, so even if they are less efficiently taken up into the protoplasts, there is more available at the protoplast cell wall to be taken up. The cellulosic nature of HPMCAS may promote active trafficking into cells in a manner that remains to be determined.

The above point about the importance of nanomaterial surface chemical composition may also explain the difference between the results found here, in which the more highly charged PAA- and PDMAEMA-coated NCs exhibited higher whole-plant translocation than the neutral PEG-coated NCs and Zhang et al.’s findings for whole plant translocation of macromolecular star polymer carriers. Zhang et al. found that carriers with a lower magnitude of zeta potential translocated more than those with a higher magnitude, also in tomato.^9^ Several differences exist that can account for this. First, the conformation and chemistry of the surface polymer are not the same. FNP NCs stabilized with block copolymers, such as the ones used here, exhibit a densely packed polymer surface with up to 1.5 polymer chains per nm^2^ that leads to a brush conformation^20^ and are more similar to the particles used to develop the LEEP model than the macromolecular star polymers. The molecular weight of the surface coating is relatively low (∼5 kDa), such that it accounts for only about 5 nm of the 40 nm NC diameter; the rest is a condensed hydrophobic core that is not wetted. Zhang’s macromolecular star polymer carriers contained core–shell blocks that are both expected to be almost fully hydrated, with more degrees of freedom than the brush conformation polymers on the FNP NCs. The majority of the reported hydrodynamic radii of these carriers are due to these hydrated blocks. The interactions between the “soft” macromolecular star polymer and the lipid bilayers of protoplasts could reasonably be different from the less soft FNP particles, given these differences in structure and properties.^41^

The second reason for the different behaviors may be related to charge. The most neutral star polymer studied by Zhang et al. (that exhibited the highest degree of translocation in tomato) had a surface zeta potential of −12 mV, whereas the most neutral FNP NCs (that exhibited a negligible degree of translocation) had a zeta potential of only −0.3 mV. It is difficult to compare exact zeta potential magnitudes between the two particle types because neither particle is the hard sphere that the Malvern software package assumes for its Smoluchowski model to calculate zeta potential.^42^ However, FNP NCs have a significant condensed-phase core, as described above, which is more consistent with the Smoluchowski model than the macromolecular star polymers. In any case, the reported >∼−10 mV “apparent” zeta potential of Zhang’s PAA-*b*-PMSEA star polymer carriers may have been large enough to promote uptake into protoplast cells.

A third difference is that on Zhang’s (PAA-*b*-P(MSEA-*co*-MTEA)) star polymer carriers, hydrophobic interactions between PMTEA blocks could have given rise to self-assembled hydrophobic patches at or near the surface, which could reasonably be expected to lead to a different protein corona adsorption profile once in the mesophyll. The effect of protein corona composition on nanomaterial transport in plants is currently poorly understood and remains an active area of research.^43^ If lessons learned in the human drug delivery protein corona literature are analogous to those of plants, differences in composition may significantly affect trafficking in vivo.

To summarize this comparison, there are more factors to consider than nanomaterial size and zeta potential when looking at translocation. Surface properties such as molecular structure, polymer conformation, and protein corona adsorption profiles, as well as the deformability, density, and degree of hydration of the whole carrier may all matter, and more research is needed to elucidate these interactions at the molecular scale.

**Figure 4:**
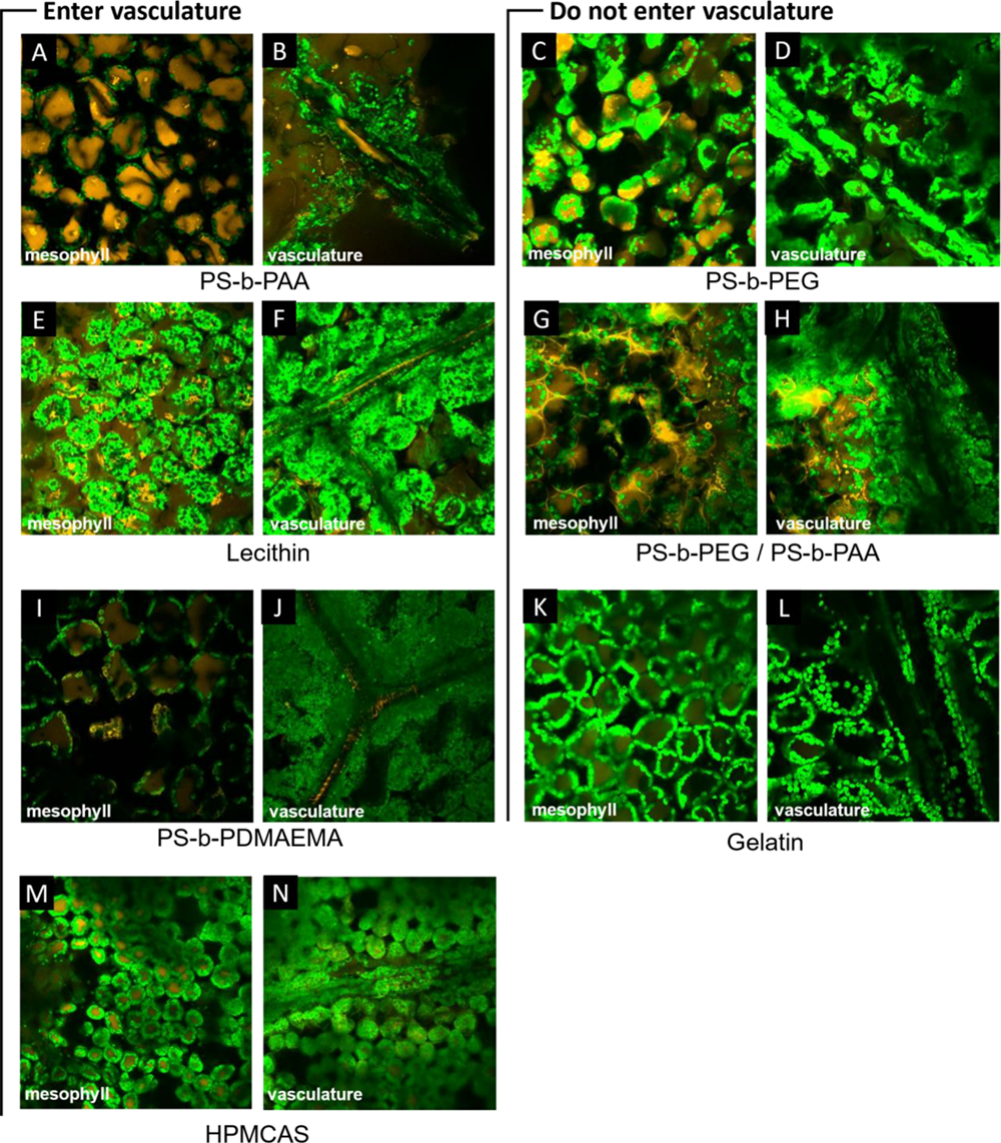
(A–N) Fluorescent confocal microscopy images of leaves dosed with NCs. NCs are colored orange, and chloroplasts are shown in green. Results suggest phloem loading for NCs coated with HPMCAS (see also Figure S3 for a clearer picture of the NP signal), PS-*b*-PAA, PS-*b*-PDMAEMA, and lecithin NCs but not PS-*b*-PEG + PS-*b*-PAA, PS-*b*-PEG, or gelatin.

The translocation results based on the presence of Gd presented in Figure 3 were corroborated by the confocal microscopy results in Figure 4, and synchrotron XRF mapping results are shown in Figure S1. NCs that exhibited significant translocation—those coated with HPMCAS, PS-*b*-PAA, PS-*b*-PDMAEMA, and lecithin—also appear to exhibit a high degree of cellular internalization and can be seen in the vascular regions of the leaf (phloem) (Figure 4A,B,E,F,I,J,M,N). The behavior of the neutral PS-*b*-PEG + PS-*b*-PAA NCs, which did not exhibit high translocation, is noticeably absent from the vascular regions. NCs appear to outline mesophyll cells with low cellular internalization (Figure 4G). Correspondingly, no NCs are visible in the vasculature (Figure 4H, right).

## Conclusions and Future Prospects

The results presented here demonstrate that the FNP platform can be used to prepare organic core–shell NCs for efficient uptake and systemic biodistribution of agrochemicals to plants following foliar administration. NCs were internalized into the leaves with a high degree of efficiency (up to 80%), which offers an attractive delivery route for agrochemicals that suffer from poor internalization in their bulk form. Likewise, the 32% translocation achieved by the HPMCAS-coated NCs and the 11% translocation exhibited by NCs coated with PS-*b*-PAA and PS-*b*-PDMAEMA outstrips by more than an order of magnitude the <1% systemic translocation reported for many foliarly applied agrochemicals in the literature.^4,7^

Trends in the translocation results for the FNP particles generally followed predictions for nanomaterial uptake through lipid membranes made by the LEEP model, namely, both size and surface charge were significant factors in governing the uptake. More highly charged NCs translocated to a greater degree than neutral NCs of the same size, and smaller NCs translocated more than larger NCs of the same surface charge. The HPMCAS formulation was an exception to this and demonstrates the need to understand how the specific chemical composition of the shell materials affect transport. Likewise, the kinetics of uptake and translocation remain to be explored more fully and are the subject of future work.

The scalability,^44^ tunability,^17^ and flexibility of the FNP platform make it an attractive technique for the development of next-generation agrochemical formulations. In particular, the core and shell of FNP NCs are independent of one another, and the NC particle surface properties are a function of the stabilizing shell material only. This suggests that once the optimal NC surface properties for foliar internalization, phloem loading, and/or translocation have been determined, it will be straightforward to prepare NC formulations that both (1) encapsulate the core material(s) of interest and (2) have the optimal shell properties for the desired movement. Future work will focus on optimizing these properties for NC uptake and movement across various agrochemical applications and across plant species.

## Supporting Information Available

Detailed feed stream information for each FNP particle synthesis; method used for organic solvent removal after particle synthesis; composition of the SAF used for characterization and metal leaching studies; synchrotron X-ray fluorescence mapping method details and maps for selected particle types; root dosing procedure; properties of different FNP particles; detailed results of NC translocation and leaf internalization; NC translocation and mass balances for root-applied NCs containing Eu; and hyperspectral library and additional confocal microscopy images (PDF)
