# Supplementary material for: Flash NanoPrecipitation as an Agrochemical Nanocarrier Formulation Platform: Phloem Uptake and Translocation after Foliar Administration
Source: ACS Agric Sci Technol. 2023 Oct 17;3(11):987–95. doi: 10.1021/acsagscitech.3c00204 (PMC10664067; doi:10.1021/acsagscitech.3c00204)
Supplement: Supplementary file 1 — as3c00204_si_001.pdf [file as3c00204_si_001.pdf]

Supplemental Information for

Flash NanoPrecipitation as an agrochemical nanocarrier formulation platform: phloem uptake and translocation after foliar administration

Kurt Ristroph[1,2], Yilin Zhang[1], Valeria Nava[1], Jonas Wielinski[1], Hagay Kohay[1], Andrew M. Kiss[3], Juergen Thieme[3], Gregory V. Lowry[1]

1. Department of Civil and Environmental Engineering, Carnegie Mellon University, Pittsburgh, PA 15213, USA.

2. Current address: Department of Agricultural and Biological Engineering, Purdue University, West Lafayette, IN 47907, USA

3. NSLS-II, Brookhaven National Laboratory, Upton, NY 11973-5000, USA

Methods

Feed streams to FNP

Table S1 – compositions of FNP feed streams for NC formulations.

Stabilizer	Organic stream (THF)	Water stream	Reservoir
50 / 50<br>PS-PEG / PS-PAA	0.833 mg/mL Gd colloids<br>1.607 mg/mL PS<br>0.5 mg/mL Vitamin E Ac<br>0.06 mg/mL 762 dye<br>1.5 mg/mL PS-PEG<br>1.5 mg/mL PS-PAA	25 mM KCl 10 mM sodium acetate, pH 5.5	4 mL water
PS-PAA	0.833 mg/mL Gd colloids<br>1.607 mg/mL PS<br>0.5 mg/mL Vitamin E Ac<br>0.06 mg/mL 762 dye<br>3 mg/mL PS-PAA	25 mM KCl 10 mM sodium acetate, pH 5.5	4 mL water
PS-PDMAEMA	0.833 mg/mL Gd colloids<br>1.607 mg/mL PS<br>0.5 mg/mL Vitamin E Ac<br>0.06 mg/mL 762 dye<br>3 mg/mL PS-PDMAEMA	25 mM KCl 10 mM sodium acetate, pH 5.5	4 mL water
Lecithin	0.833 mg/mL Gd colloids<br>0.793 mg/mL PS<br>0.333 mg/mL Vitamin E Ac<br>0.04 mg/mL 762 dye<br>4 mg/mL Lecithin	25 mM KCl 10 mM sodium acetate, pH 5.5	4 mL water
PS-PEG	0.833 mg/mL Gd colloids<br>1.607 mg/mL PS<br>0.5 mg/mL Vitamin E Ac<br>0.06 mg/mL 762 dye<br>3 mg/mL PS-PEG	25 mM KCl 10 mM sodium acetate, pH 5.5	4 mL water
Gelatin	0.833 mg/mL Gd colloids<br>0.793 mg/mL PS<br>0.333 mg/mL Vitamin E Ac<br>0.04 mg/mL 762 dye	4 mg/mL gelatin	4 mL water

Stabilizer	Organic stream 1 (THF)	Organic stream 2 (THF)	Water streams, 2x	Reservoir
HPMCAS-126	1.389 mg/mL Gd colloids<br>0.644 mg/mL PS<br>0.416 mg/mL Vit E<br>0.05 mg/mL 762 dye	7.5 mg/mL HPMCAS-126	25 mM KCl 10 mM sodium acetate, pH 5.5	8 mL water

Note: all formulations were prepared using a two-inlet CIJ mixer except the HPMCAS-stabilized formulation, which was prepared using the four-inlet MIVM mixer. This is because flocculation was observed when HPMCAS was co-loaded in a THF stream with Gd colloids.

Organic solvent removal

Following FNP, residual organic solvent was removed by either dialysis or rinsing by centrifugal ultrafiltration. In the former, 5mL of NC suspension were dialyzed against 500 mL of deionized water for at least 4 hours using 6-8k MWCO dialysis tubing (Thermo Fisher Scientific). In the latter (used for NC formulations with a neutral zeta potential, to reduce NC mass losses to dialysis tubing), 1mL of deionized water was added to 1mL of NC suspension; the suspension was rinsed on a 3mL 100kDa MWCO Amicon centrifugal ultrafiltration cartridge at 5,000g for 15 minutes to reduce the volume to 1mL; the process was repeated three more times to reduce the organic solvent content below 1%. These washed NCs were used for all subsequent tests.

SAF composition

The composition of simulated apoplastic fluid used as a dialysis medium to determine metal leaching from NCs was replicated from Table S2 of Lohaus G, Pennewiss K, Sattelmacher B, Hussmann M, Hermann Muehling K. Is the infiltration-centrifugation technique appropriate for the isolation of apoplastic fluid? A critical evaluation with different plant species. *Physiol Plant.* 2001 Apr;111(4):457-465. doi: 10.1034/j.1399-3054.2001.1110405.x. PMID: 11299010.

X-ray fluorescence mapping

X-ray fluorescence mapping (XRF) images with submicron resolution of lyophilized tomato leaf cross-sections were acquired at beamline 5-ID at the National Synchrotron Light Source II (NSLS-II) at Brookhaven National Laboratory.[1] 24h after foliar application of NCs, the dosed leaves were harvested, cut, submerged in Tissue-Tek® O.C.T. Compound, and immediately flash frozen in liquid nitrogen. The tissues were cut into 50 μm thick slices perpendicular to the leaf vasculature using a cryostat. Each cross-section was mounted onto a polystyrene cell culture slide sealed between two pieces of Kapton tape. The cross-sections were kept frozen until lyophilization at -80 °C and 0.133 mbar for 48 h. To acquire XRF

images, samples were oriented at a 45° angle to the incoming beam (0.5 µm x 0.5 µm, 9.5 KeV) and to a four-element Vortex ME3 silicon drift detector. High-resolution XRF maps were collected using a step size of 0.5 µm on a selected location on the fixed tissue using a dwell time of 0.25 s. The spectral fitting was performed using the PyXRF spectral fitting program.[2]

Root dosing

A suite of NCs with the same sizes and surface chemistries as in the main body text was prepared encapsulating Eu as a tracer metal instead of Gd. This suite of NCs was dosed to plant roots instead of leaves, by spiking 0.05mL of NC solution directly into the hydroponic Hoagland's solution of 4-week old Roma tomato seedlings (n=5 plants per formulation). After 72h, plants were sectioned as described in the body, and Eu was measured in plant tissue.

Results

Nanocarrier formulation and characterization

Table S2 – NC size, PDI, zeta potential, and Gd content after organic solvent removal. The final column was calculated by dividing the mass of Gd measured in bulk SAF after 72h of dialysis by the initial mass of Gd added into the dialysis bag. NC size was measured in deionized water at pH 6 and zeta potential was measured in 15 mM NaCl at pH 6.

**Stabilizer**	**Z-average size (nm)**	**PDI**	**Zeta potential (mV)**	**Core loading**	**Gd content (ppm)**	**Gd loss to bulk SAF solution over 72h**
PS-b-PAA	55 ± 1	0.25 ± 0.01	-39.6 ± 3.6	50%	14.1	0.0%
PS-b-PDMAEMA	57 ± 3	0.27 ± 0.03	38.4 ± 3.3	50%	9.5	0.0%
Lecithin	184 ± 5	0.15 ± 0.02	-23.4 ± 3.5	33%	11.9	7.0%
50 / 50 PS-b-PEG / PS-b-PAA	75 ± 2	0.21 ± 0	-7.7 ± 0.9	50%	14.5	0.1%
PS-b-PEG	71 ± 1	0.17 ± 0.02	-0.3 ± 0.4	50%	5.2	0.0%
Gelatin	198 ± 4	0.11 ± 0.01	5.2 ± 0.3	33%	6.8	0.1%
HPMCAS	136 ± 5	0.14 ± 0	-10.3 ± 0.9	25%	3.7	1.4%

Nanocarrier translocation

Table S3 –Nanocarrier translocation and mass balances for foliarly-applied NCs containing Gd

NP stabilizer	Percent of theor. Gd detected	Translocation*	UP*	LO*	ST*	RT*	EX*	HG*
PS-b-PAA	104±12%	12±7%	2±1%	1±0%	6±7%	3±1%	88±7%	1±1%
PS-b-PDMAEMA	125±9%	10±3%	3±1%	1±0%	3±2%	3±1%	90±3%	0±0%
Lecithin	160±11%	7±5%	1±1%	1±1%	4±4%	2±1%	93±5%	0±0%
PS-b-PEG / PS-b-PAA	154±7%	2±2%	0±0%	0±0%	1±2%	0±0%	98±2%	0±0%
PS-b-PEG	76±12%	2±2%	0±0%	0±0%	1±2%	0±0%	97±3%	1±1%
Gelatin	58±2%	8±7%	1±0%	1±1%	4±5%	1±1%	92±7%	1±0%
HPMCAS	38±3%	32±12%	4±4%	2±1%	17±11%	8±3%	68±12%	1±1%
Gd control	170±9%	2±2%	0±0%	0±0%	1±1%	1±1%	98±2%	0±0%

Translocation = (mass of Gd in UP, LO, ST, RT, HG) / (total mass of Gd measured)
* - normalized by total measured Gd in sample

For NCs stabilized by HPMCAS, PEG, and gelatin, an approximate Hoagland solution volume of 40mL was used for calculations of metal content in that section.

Nanocarrier internalization

Table S4 –Nanocarrier internalization and mass balances for foliarly-applied NCs containing Gd

NP stabilizer	Percent of theor. Gd detected	Internalization*
PS-b-PAA	153±18%	79±9%
PS-b-PDMAEMA	168±8%	75±6%
Lecithin	84±9%	83±3%
PS-b-PEG / PS-b-PAA	126±10%	69±3%
PS-b-PEG	39±9%	53±3%
Gelatin	61±19%	75±5%
HPMCAS	22±6%	55±6%
Gd control	148±45%	60±10%

* - normalized by total measured Gd in sample

Synchrotron X-ray fluorescence mapping

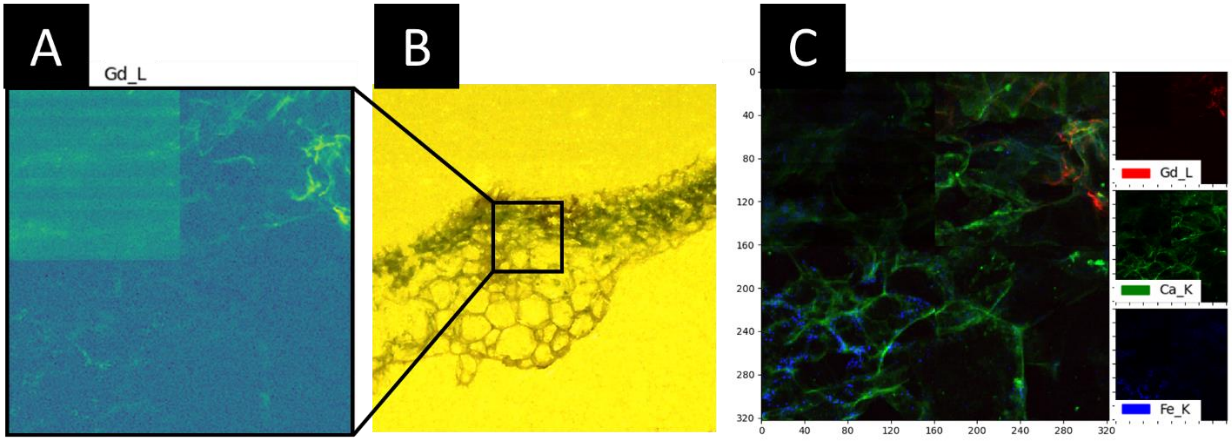

Figure S1. Synchrotron XRF mapping results on a tomato leaf cross-section dosed with PS-b-PAA-coated NCs containing Gd. An optical image of the cross section is shown in (B), with the boxed region approximating the mapped regime. (A) and (C) show maps for identified elements Gd (A, and red in C), Ca (green in C), and Fe (blue in C). Visible in (A) is a small amount of Gd detected in the vascular bundle (phloem) region of the cross-section, which corroborates the translocation and confocal microscopy results from the main text.

Root dosing

Table S5 – Nanocarrier translocation and mass balances for root-applied NCs containing Eu.

NP shell layer	Percent of theor. Eu detected	Uptake*	RT*	HG*
PS-b-PAA	57±20%	2±1%	60±25%	38±25%
PS-b-PDMAEMA	136±62%	2±2%	50±22%	48±24%
Lecithin	79±13%	1±0%	73±11%	26±12%
PS-b-PEG / PS-b-PAA	62±24%	2±1%	67±24%	31±25%
Eu control	116±22%	2±1%	44±15%	54±15%

Uptake = (mass of Eu in UP, LO, ST, EX) / (total mass of Eu measured)
* - normalized by total measured Eu in sample

Root uptake summary: effectively no NC uptake through the roots was observed, in line with expectations from literature.

Hyperspectral library development and hyperspectral imaging

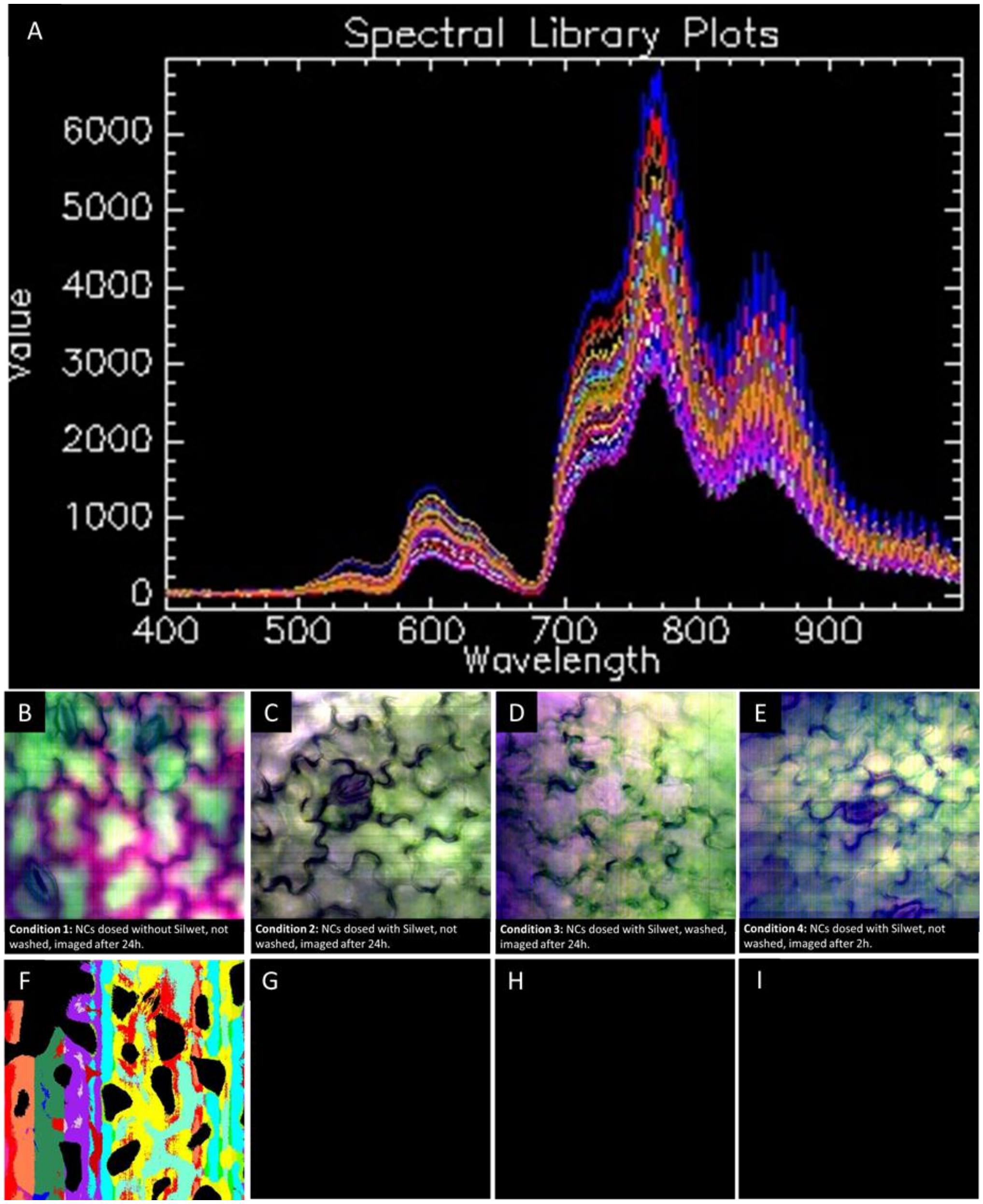

Figure S2: (A) Spectral library plots for PAA-coated NCs that were used to develop Figure 4 and sections F, G, H, and I.
Sections B and H show epidermis of leaves dosed with NCs, without Silwet, 24h before imaging. These measurements were used to develop the NC hyperspectral library shown in A.
Sections C and G show leaves dosed with NCs and Silwet 24h prior to imaging (no wash). Sections D and H show leaves dosed with NCs and Silwet 24h before imaging (leaves washed prior to imaging). Sections E and I show leaves dosed with NCs and Silwet 2h before imaging (no wash prior to imaging).

No NC signature was detected in either of the latter three conditions (n=4), suggesting a high degree of NC internalization.

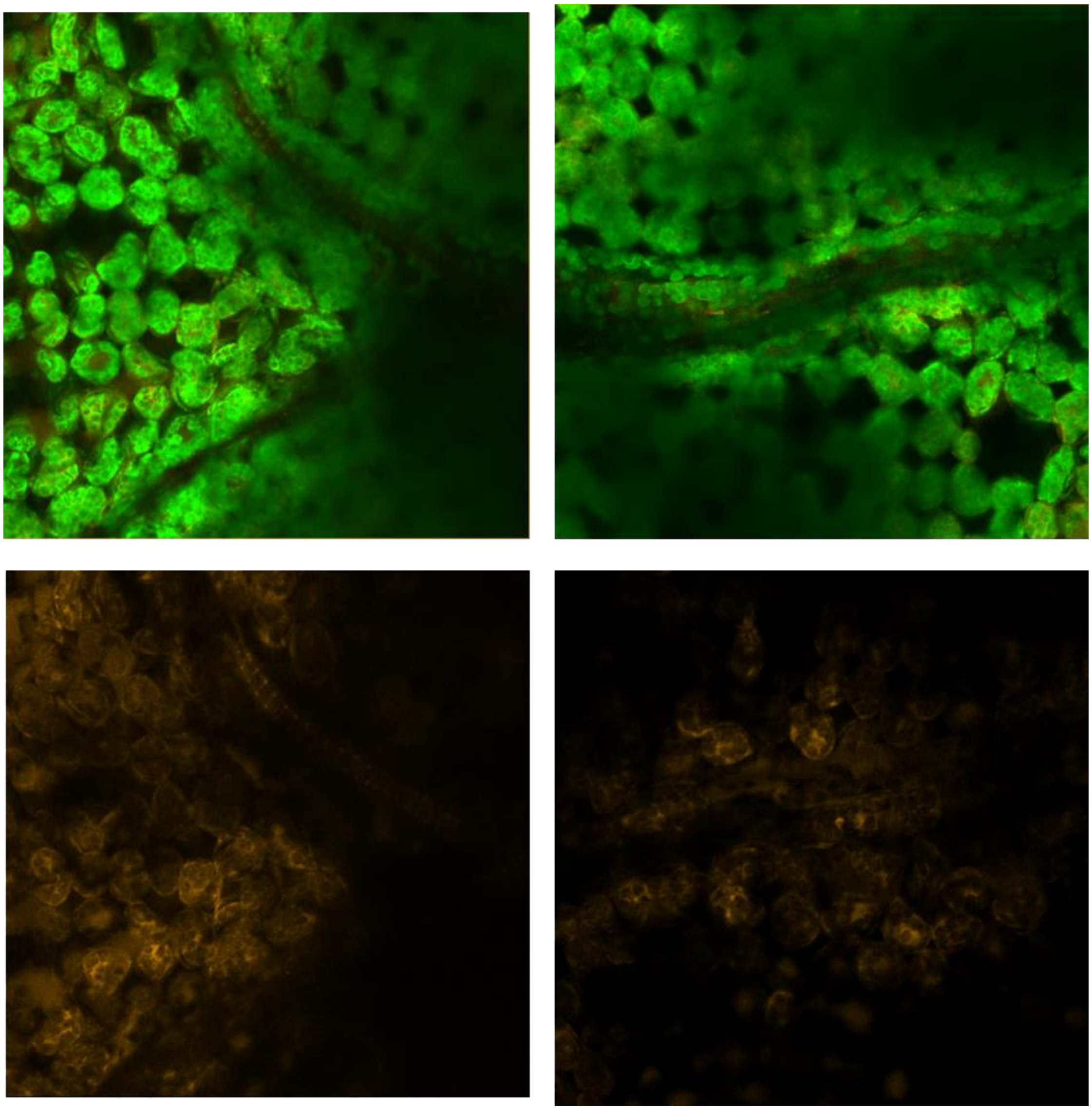

Figure S3: Fluorescent confocal microscopy images of leaves dosed with HPMCAS-coated NCs. In the bottom images, only the orange channel can be seen to emphasize the presence of NCs in the vasculature.

References

(1) Nazaretski, E.; Coburn, D. S.; Xu, W.; Ma, J.; Xu, H.; Smith, R.; Huang, X.; Yang, Y.; Huang, L.; Idir, M.; et al. A new Kirkpatrick-Baez-based scanning microscope for the Submicron Resolution X-ray Spectroscopy (SRX) beamline at NSLS-II. *Journal of Synchrotron Radiation* **2022**, *29* (5), 1284-1291. DOI: doi:10.1107/S1600577522007056.
(2) Li, L.; Yan, H.; Xu, W.; Yu, D.; Heroux, A.; Lee, W.-K.; Campbell, S.; Chu, Y. *PyXRF: Python-based X-ray fluorescence analysis package*; SPIE, 2017.
